# Percutaneous Pedicle Screw Fixation with Polymethylmethacrylate Augmentation for the Treatment of Thoracolumbar Intravertebral Pseudoarthrosis Associated with Kummell's Osteonecrosis

**DOI:** 10.1155/2016/3878063

**Published:** 2016-08-10

**Authors:** Hyeun-Sung Kim, Dong-Hwa Heo

**Affiliations:** ^1^Department of Neurosurgery, Spine Center, Nanoori Hospital, Suwon 16503, Republic of Korea; ^2^Department of Neurosurgery, Spine Center, The Leon Wiltse Memorial Hospital, Suwon 16480, Republic of Korea

## Abstract

*Purpose*. The purpose of our study is to evaluate the therapeutic efficacy of short-segment percutaneous pedicle screw fixation with polymethylmethacrylate (PMMA) augmentation for the treatment of osteoporotic thoracolumbar compression fracture with osteonecrosis.* Methods*. Osteoporotic thoracolumbar compression fractures with avascular necrosis were treated by short-segment PPF with PMMA augmentation. Eighteen were followed up for more than 2 years. The kyphotic angle, compression ratio, visual analog scale (VAS) score for back pain, and the Oswestry Disability Index (ODI) were analyzed. In addition, radiologic and clinical parameters of PPF group were compared with percutaneous vertebroplasty (PVP) group.* Results*. Vertebral height and kyphotic angle of the compressed vertebral bodies were significantly corrected after the operation (*P* < 0.05). Further, restored vertebral height was maintained during the 2 or more years of postoperative follow-up. Compared to the PVP group the postoperative compression ratio and kyphotic angle were significantly lower in the PPF group (*P* < 0.05). The postoperative ODI and VAS of the PVP group were significantly higher than the PPF (*P* < 0.05).* Conclusions*. According to our results, short-segment PPF with PMMA augmentation may be an effective minimally invasive treatment for osteoporosis in cases of osteoporotic vertebral compression fractures with Kummell's osteonecrosis.

## 1. Introduction

Occasionally, noninfected avascular osteonecrosis is detected in osteoporotic vertebral body compression fractures. Intravertebral avascular osteonecrosis presented fluid collection or the presence of conjunction with air cleft in compressed vertebral body. A necrotic cavity can develop intravertebral pseudoarthrosis and result in spinal instability. Percutaneous vertebroplasty or kyphoplasty using polymethylmethacrylate (PMMA) has been attempted to treat compression fractures with avascular necrosis [1–6]. Several studies have reported that vertebroplasty to treat an osteoporotic vertebral compression fracture with osteonecrosis is highly effective [1–5]. However, these studies reported short-term clinical and radiological results within 12 months. Long-term clinical and radiologic results of percutaneous vertebroplasty or kyphoplasty were poor [6, 7]. Percutaneous vertebroplasty may not be supportive enough for the long-term stabilization effect. Spinal fusion operations have been performed in Kummell's osteonecrosis; however, extensive fusion operations have morbidities and complications associated with surgical procedures and long surgical time. In our study, we have tried to perform short-segment pedicle screw fixation with PMMA augmentation after postural reduction. The purpose of this study was to assess, for at least 2 years, the radiologic and clinical outcomes of patients who underwent short-segment pedicle screw fixation with PMMA augmentation to treat osteoporotic vertebral compression fractures with intravertebral pseudoarthrosis comparing to patients who were treated by percutaneous vertebroplasty.

## 2. Materials and Methods

The current study was designed as a retrospective review of clinical and radiologic parameters. From October 2010 to March 2013, 21 patients who had osteoporotic compression fractures with avascular osteonecrosis were treated by short-segment percutaneous pedicle screw fixation (PPF) with PMMA augmentation in two hospitals in which the authors were affiliated. And 45 patients were treated by percutaneous vertebroplasty (PVP). We only included 49 patients who had a single-level osteoporotic vertebral compression fracture with avascular osteonecrosis and were able to be followed for more than 24 months.

Before the operation, we took dynamic X-ray films, measured bone mineral density, and performed magnetic resonance imaging (MRI) and computed tomography (CT) to define acute osteoporotic compression fractures with avascular osteonecrosis and intravertebral air cleft in all the patients. Intravertebral pseudoarthrosis or instability associated with Kummell's osteonecrosis was identified with dynamic X-ray: anterior vertebral height and kyphotic angulation are changed on lateral flexion and extension views. Vertebral plana and irreducible collapsed vertebrae with a small portion of the vacuum cleft were excluded. Also, we excluded the patients who had clinical histories of bone metabolic disorders, primary bone tumors, or malignancies including metastasis.

18 patients who were treated by PPF were assigned to “PPF group” and 31 patients who were treated by PVP were assigned to “PVP group.”

We performed this investigation in accordance with our institutional guidelines, which comply with international laws and policies (⁎⁎⁎⁎ Hospital Institutional Review Board, #2014-03).

### 2.1. Surgical Procedures of Short-Segment Fixation with Augmentation [8]

**Figure 1:**
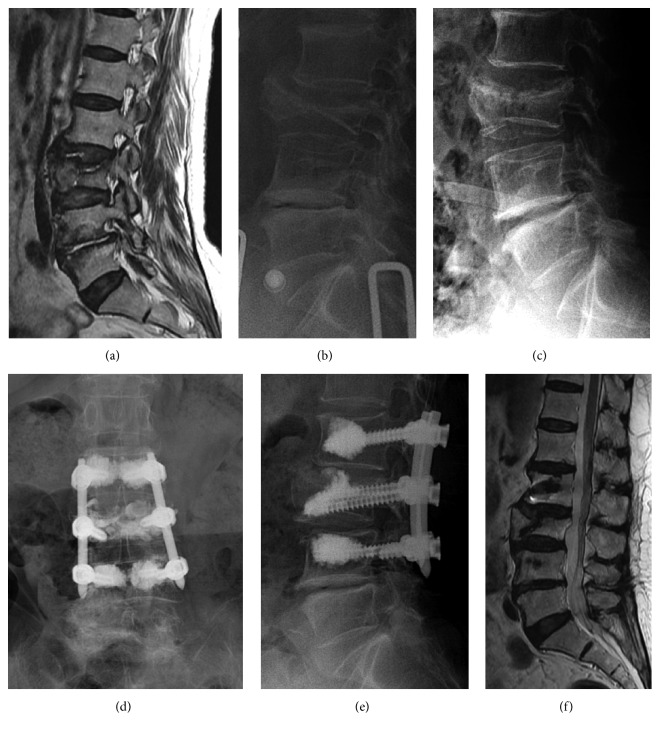
Preoperative MRI and X-ray show a L3 compression fracture with osteonecrosis ((a) and (b)). The compressed L3 body was reexpanded after postural reduction (c). The compressed vertebral body with osteonecrosis was well healed and still restored in spite of left L4 pedicle screw fracture at the 24-month follow-up ((d), (e), and (f)).

First, all enrolled patients had received conservative management such as bed rest, analgesics, and brace wearing for at least 2 weeks after diagnosis of Kummell's osteonecrosis. Despite conservative management, patients presented intractable pain or progression of vertebral body compression and we planned surgical treatments. All the patients received postural reduction 24 hours before the operation. All surgical procedures were performed in the prone position under epidural or endotracheal anesthesia. PMMA was injected bilaterally via transpedicular trajectory just before insertion of the percutaneous pedicle screw under C-arm fluoroscopic guidance. Percutaneous pedicle screw insertion and PMMA augmentation was performed on the fractured vertebral body as well as 1 level above and below (Figure 1). If the patient had an L2 compression fracture with osteonecrosis, we inserted PMMA and percutaneous pedicle screws at L1, L2, and L3. Finally, two percutaneous rods and screw caps were applied. We did not perform additional posterolateral fusion procedures such as an autologous iliac bone graft or an allofusion material graft. If patients had neurologic deficit symptoms with thecal sac compression by retropulsed bony fragments and acute multilevel compression fractures, we did not perform these operative treatments.

### 2.2. Analysis of Clinical Parameters

We retrospectively reviewed the preoperative clinical parameters such as age, gender, bone mineral density, visual analog scale (VAS) score for back pain, and the Oswestry Disability Index (ODI). We checked the VAS score of back and ODI preoperatively and postoperatively at 7 days, 12 months, and 24 months or more (the final follow-up period). We compared the preoperative VAS scores and ODI with the immediate postoperative scores and also compared the immediate postoperative VAS scores and ODI with the VAS score and ODI at 12 months after the operation and at the final follow-up period after operations. We also reviewed operation time, bleeding amount, and complication related to anesthesia and operation procedures.

### 2.3. Analysis of Radiological Parameters

The presence of avascular osteonecrosis in the vertebral body was defined as the collection of intravertebral fluid or the presence of conjunction with air, as seen via MRI and CT scan. A CT scan finding of intravertebral vacuum phenomenon was defined as an area of air density in the fractured vertebral body. We reviewed radiological parameters, such as the compression ratio and kyphotic angle.

We reviewed serial follow-up plain radiographs immediately after the operation and postoperatively at 12 months and approximately 24 months (the final follow-up period). The anterior and posterior heights of the fractured vertebral body with avascular osteonecrosis were assessed in order to calculate the compression ratio (anterior/posterior height; AP ratio) before and after the operation. The kyphotic angle was measured using an angle between the lower endplate of the upper vertebral body and the lower endplate of the affected vertebral body.

The degree of compression progression of the cemented compressed vertebral bodies, which are the compression ratio and kyphotic angle difference between the immediate postoperative measurement and the final follow-up period measurements, was calculated for all of the patients. We compared each of the compression ratio and kyphotic differences. We also performed CT or MRI for the evaluation of bone healing in the fractured vertebral bodies during the final follow-up period.

### 2.4. Comparative Analysis with Percutaneous Vertebroplasty Group

Forty-five patients received percutaneous vertebroplasty with PMMA for the treatment of osteoporotic compression fracture with osteonecrosis. Among them, thirty-one patients were followed for more than 2 years. We compared radiological parameters such as kyphotic angle and compression ratio of PPF group with PVP group. We compared the mean differences in the compression ratios of the cemented vertebral bodies and mean kyphotic angles between the “PPF group” and the “PVP group.”

Additionally, we compared clinical results such as ODI and VAS score of back of PPF group with PVP group.

### 2.5. Statistical Analysis

Statistical analysis was performed using the Mann-Whitney* U* test and the Wilcoxon rank sum test. The level of significance was set at 0.05. SPSS 12.0 for Windows (SPSS, Chicago, IL, USA) was used for the statistical analysis.

## 3. Results

The mean age of PPF group was 69.5 ± 5.1 years (15 females and 3 males). The mean follow-up period was 24.8 ± 1.3 months (24–27 months). The treated levels were distributed from T12 to L3 with five in T12, nine in L1, three in L2, and one in L3. The mean T-score of the bone mineral density was −3.55 ± 0.59. The mean volume of estimated blood loss was 91.1 ± 27.6 mL, and the mean operation time was 64.7 ± 15.4 minutes (Table 1). The mean age of PVP group was 71.1 ± 3.9 (21 females and 9 males). The treated levels were distributed from T11 to L3. There were no differences between PPF group and PVP group (Table 1).

### 3.1. Compression Ratio and Kyphotic Angle Changes after Short-Segment Fixation with Augmentation

**Figure 2:**
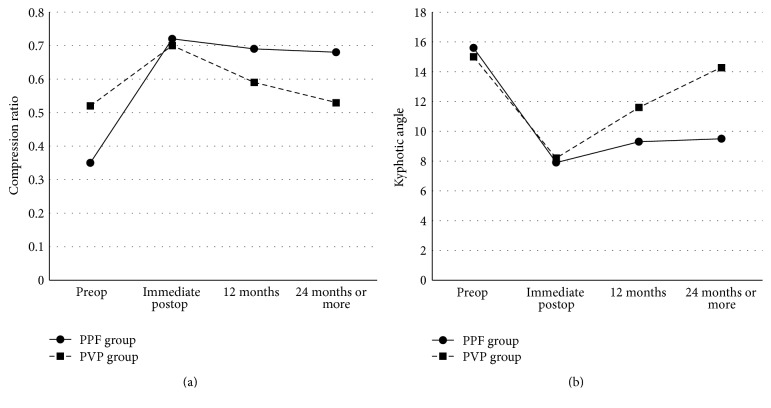
Serial changes in the compression ratio (a) and kyphotic angle (b). The PPF group maintained height of the compressed vertebral bodies after the operation compared to the PVP group.

The vertebral height and kyphotic angle of the compressed vertebral bodies were significantly corrected from 0.35 ± 0.12 to 0.72 ± 0.08 and from 15.56 ± 3.69° to 7.90 ± 2.65° after operation in PPF group (*P* < 0.05). Although restored vertebral height was slightly decreased 1 year after the operation the compression ratio and kyphotic angle were well-maintained during the approximately 2 years of postoperative follow-up. The immediate postoperative mean compression ratio was 0.72 ± 0.08 and slightly decreased to 0.69 ± 0.07 at 1-year follow-up and 0.68 ± 0.07 at the 2-year follow-up (Figure 2(a)). The immediate postoperative mean kyphotic angle was 7.90 ± 2.65° and slightly increased to 9.28 ± 2.53° at the 1-year follow-up and 9.56 ± 2.64 at the 2-year follow-up (Figure 2(b)).

Bone healing of the compression fracture with osteonecrosis area was detected on finally followed CT or MRI images in all enrolled patients. Intravertebral fluid signal of MRI or intravertebral air density (vacuum phenomenon) of CT has completely disappeared (Figure 1(f)).

### 3.2. Comparative Analysis with Percutaneous Vertebroplasty Group

There were 31 patients in the vertebroplasty group and 18 patients in pedicle screw group. The mean difference between the compression ratios taken immediately postoperative and during the final follow-up period was 0.04 ± 0.03 in PPF group and 0.18 ± 0.08 in the PVP group. The mean difference in compression ratios of the PVP group was significantly higher than that of the PPF group (*P* < 0.05, Table 2). The mean difference in measurements of the kyphotic angle taken immediately postoperative and during the final follow-up period was 1.65 ± 1.22° in the PPF group and 6.06 ± 3.38° in the PVP group. The mean difference of the kyphotic angle of the PVP group was significantly higher than that of PPF group (*P* < 0.05, Table 2). The postoperative compression ratio of the PPF group was significantly higher than the PVP group, and the kyphotic angle of the PPF group was significantly lower than PVP group (Figures 2(a) and 2(b)).

**Figure 3:**
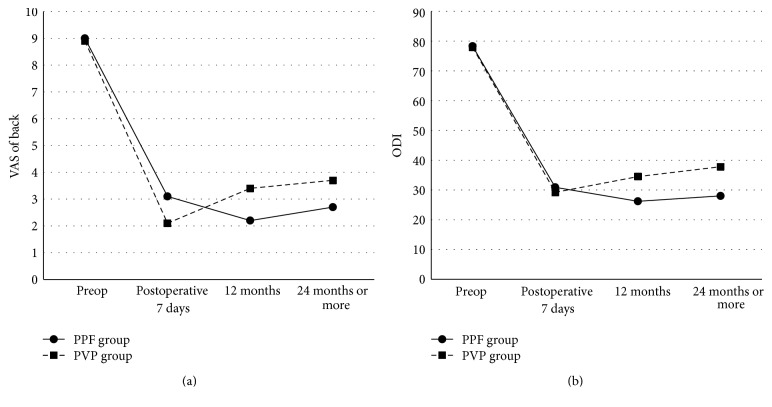
Serial changes in VAS (a) and the ODI (b). The ODI and VAS score of the PVP group are significantly higher than the PPF group at the final follow-up appointment (postoperative 24 months or more).

Preoperative ODI and VAS score were not significantly different in both groups. The mean preoperative VAS score was 9.00 ± 0.84 and 7 days post operation it was 3.11 ± 1.37, indicating that the mean VAS score decreased significantly after the initial operation (*P* < 0.05) in PPF group. VAS scores measured during the postoperative follow-up showed that the mean VAS scores were 2.17 ± 0.86 at 12-month follow-up and 2.67 ± 1.03 at the final follow-up appointment (Figure 3(a)) in PPF group. The mean ODI was 78.28 ± 3.85 preoperatively, 30.89 ± 5.87 at 1 day after the operation, 26.17 ± 6.53 at 12-month follow-up, and 28.00 ± 5.48 at the final follow-up appointment in PPF group. The mean ODI significantly decreased after the operation (*P* < 0.05, Figure 3(b)).

In PVP group, the mean preoperative VAS score was 8.90 ± 0.83 and 7 days after operation it was 2.06 ± 0.85, indicating that the mean VAS score decreased significantly after the initial operation (*P* < 0.05). Preoperative mean ODI was significantly decreased from 77.90 ± 1.83 to 29.19 ± 4.17 at 7 days after the operation in PVP group (*P* < 0.05, Figure 3(b)). The mean VAS score was increased from 2.06 ± 0.85 to 3.71 ± 1.27 at the final follow-up appointment in PVP group. And the mean ODI was increased from 29.19 ± 4.17 to 37.77 ± 11.06 at the final follow-up appointment in PVP group. The postoperative ODI and VAS scores of the PVP were significantly higher than the PPF group at the final follow-up period (Figures 3(a) and 3(b), Table 2).

### 3.3. Comparative Analysis with Percutaneous Vertebroplasty Group

There were 31 patients in the vertebroplasty group and 18 patients in pedicle screw group. The mean difference between the compression ratios taken immediately after operation and during the final follow-up period was 0.04 ± 0.03 in PPF group and 0.18 ± 0.08 in the PVP group. The mean difference in compression ratios of the PVP group was significantly higher than that of the PPF group (*P* < 0.05, Table 2). The mean difference in measurements of the kyphotic angle taken immediately after operation and during the final follow-up period was 1.65 ± 1.22° in the PPF group and 6.06 ± 3.38° in the PVP group. The mean difference of the kyphotic angle of the PVP group was significantly higher than that of PPF group (*P* < 0.05, Table 2). The postoperative compression ratio of the PPF group was significantly higher than the PVP group, and the kyphotic angle of the PPF group was significantly lower than PVP group (Figures 2(a) and 2(b)).

Preoperative ODI and VAS score were not significantly different in both groups. The mean preoperative VAS score was 9.00 ± 0.84 and 7 days after operation it was 3.11 ± 1.37, indicating that the mean VAS score decreased significantly after the initial operation (*P* < 0.05) in PPF group. VAS scores measured during the postoperative follow-up showed that the mean VAS scores were 2.17 ± 0.86 at 12-month follow-up and 2.67 ± 1.03 at the final follow-up appointment (Figure 3(a)) in PPF group. The mean ODI was 78.28 ± 3.85 preoperatively, 30.89 ± 5.87 at 1 day after the operation, 26.17 ± 6.53 at 12-month follow-up, and 28.00 ± 5.48 at the final follow-up appointment in PPF group. The mean ODI significantly decreased after the operation (*P* < 0.05, Figure 3(b)).

In PVP group, the mean preoperative VAS score was 8.90 ± 0.83 and 7 days after operation it was 2.06 ± 0.85, indicating that the mean VAS score decreased significantly after the initial operation (*P* < 0.05). Preoperative mean ODI was significantly decreased from 77.90 ± 1.83 to 29.19 ± 4.17 at 7 days after the operation in PVP group (*P* < 0.05, Figure 3(b)). The mean VAS score was increased from 2.06 ± 0.85 to 3.71 ± 1.27 at the final follow-up appointment in PVP group. And the mean ODI was increased from 29.19 ± 4.17 to 37.77 ± 11.06 at the final follow-up appointment in PVP group. The postoperative ODI and VAS scores of the PVP were significantly higher than the PPF group at the final follow-up period (Figures 3(a) and 3(b), Table 2).

In vertebroplasty group, two patients required aggressive decompression and fusion surgery due to recollapsed vertebral body and compression of the thecal sac. Two patients presented weakness in both legs. We performed anterior corpectomy for the recollapsed vertebral body and posterior pedicle screw fixation.

### 3.4. Complications Related Anesthesia and Operation

Postoperative atelectasis developed in 2 patients in the PPF group. Atelectasis was subclinical and improved immediately after ambulation. A screw fracture occurred in one patient; however, recollapse of the vertebral body did not develop, and healing of osteonecrosis was detected at the final follow-up MRI (Figure 2). In one patient, lower end pedicle screws were slightly pulled out 3 months after the operation. However, instrument failure was not aggravated until the final follow-up period. Minor stich abscess developed in one case. A new remote new vertebral compression fracture occurred in one patient. We did not experience any adjacent vertebral compression fractures or deep wound infections.

Severe recollapse of augmented vertebral bodies and spinal canal encroachment by bony fragment occurred in two patients of PVP group. Two patients required aggressive decompression and fusion surgery due to recollapsed vertebral body and compression of the thecal sac. However, there was no patient who needs additional surgical treatments in PPF group.

## 4. Discussion

Natural course of vertebral compression fractures avascular osteonecrosis named as intravertebral air cleft or pseudoarthrosis may be poor [9]. The height of the compressed vertebral bodies decreases and kyphosis is aggravated. Also, intravertebral instability has still remained above 70% [9]. Untreated intravertebral pseudoarthrosis can induce kyphotic deformity and disability. Several previous studies have reported that vertebroplasty or kyphoplasty with PMMA cement may be effective for the treatment of vertebral compression fracture with avascular osteonecrosis. Augmentation procedures with PMMA can improve refractory back pain and restore height of the compressed vertebral body height [3, 5]. However, cement augmentation procedures appear to be favorable treatments for intervertebral pseudoarthrosis only during the immediate postoperative phase and within one year [1, 3–5, 10]. At the long-term follow-up period, augmented vertebral body had recollapsed, and kyphosis had aggravated [6, 7]. Also, back pain and the ODI were aggravated during the long-term follow-up period. A recollapsed augmented vertebral body can compress neural tissue and induce neurologic deficit [6].

PMMA cannot be replaced by new bone; therefore, a foreign body reaction may occur. Fibrotic wall formation around a PMMA mass may induce micromotion and future instability [11, 12]. Sometimes, a radiolucent line named as “halo phenomenon” develops around the PMMA solid mass [13]. These phenomena may induce recollapse of the cemented vertebral body [6]. The PMMA mass has also spontaneously migrated to extravertebral lesions such as disc space or anterior vertebral space [14, 15]. Therefore, we tried additional PPF after PMMA augmentations. Vertebroplasty may not provide enough strength and stability in cases of compressed vertebrae with avascular necrosis. The most patients with intravertebral pseudoarthrosis are at an advanced age and have poor bone quality and medical illness histories; therefore, extensive fusion surgeries are hard to perform due to the length of time under anesthesia and surgical morbidities. Percutaneous pedicle screw fixation with PMMA augmentation can be performed under epidural anesthesia and has a short operation time. Furthermore, intraoperative blood loss is small, and a transfusion was not required in any of our cases. Therefore, these procedures may be compatible with older patients. However, if patients had nonreducible vertebra plana or small necrotic cavity without intravertebral pseudoarthrosis, we treated them with conservative management or vertebroplasty.

Geriatric patients have a higher incidence of complications or morbidity related to general anesthesia due to significant medical illness history. General or epidural anesthesia cannot be performed in old patients with serious medical diseases. Consequently, we performed percutaneous vertebroplasty instead. We also recommended strict activity restriction and advised the patients to wear rigid spinal orthosis for a longer period after vertebroplasty. Recently, parathyroid hormone therapy was administered to patients with intravertebral osteonecrosis.

We did not experience any major complications related to hardware failure. A screw fracture and slightly pulled-out screw developed in only two patients. Fortunately, the two patients did not develop kyphosis or recollapse of the cemented vertebral bodies, and bone healing of osteonecrosis occurred.

We suggested that osteoporotic vertebral compression fractures with osteonecrosis are a different pathologic condition compared to fractures without osteonecrosis. Therefore, vertebroplasty is a relative contraindication for Kummell's osteonecrosis. However, if the compression ratio is high and the area of the osteonecrosis is narrow, vertebroplasty may be a good treatment option.

The present study had several limitations. Our study design was not a randomized case control study but a retrospective review. In addition, we did not have a control group that underwent conservative treatments. Therefore, the results of our study cannot be generalized to all osteoporotic compression fractures with osteonecrosis. Additionally, we were only able to follow a total of 18 patients. In order to better establish the clinical and radiologic outcomes of this procedure, more patients should be studied over an extended follow-up period. Randomized case control trials should also be evaluated.

## 5. Conclusions

According to our results, short-segment percutaneous PPF with PMMA augmentation may be an effective minimally invasive treatment for osteoporosis in cases of osteoporotic vertebral compression fractures with osteonecrosis, which manifests as an intravertebral vacuum phenomenon, pseudoarthrosis, or intravertebral fluid collection during the two years or longer of patient follow-up. A short operation time, less bleeding, and no morbidity related to bone graft were maybe merit point of our treatment. However, for a more exact evaluation of the clinical and radiologic outcomes of the operation, a longer follow-up period is needed.

## Figures and Tables

**Table 1:** Characteristics of two groups.

Characteristics	PPF group	PVP group
Age (year)	69.5 ± 5.1	71.1 ± 3.9
Sex (M/F)	3/15	9/21
Bone mineral density (*T*-score)	−3.55 ± 0.59	−3.61 ± 0.57
Mean follow-up period (months)	24.8 ± 1.3	26.8 ± 2.1
Location of compression fracture	5 (T12); 9 (L1); 3 (L2); and 1 (L3)	3 (T11); 7 (T12); 15 (L1); 3 (L2); and 3 (L3)
Mean operation time	64.7 ± 15.4 minutes	
Volume of estimate blood loss	91.1 ± 15.4	
Complications related to operation	Screw fracture: 1 case	Severe recollapse of augmented vertebral body: 2 cases
	Screw minor pulled out: 1 case	

**Table 2:** Comparison of mean difference in compression ratio and kyphotic angle.

	PPF group	PVP group
Number of patients	18	31
The mean difference of AP ratio^*∗*^	0.04 ± 0.03	0.18 ± 0.08
The mean difference of kyphotic angle^*∗*^	1.65 ± 1.22	6.06 ± 3.38
VAS at postoperative 24 months^*∗*^	2.67 ± 1.03	3.71 ± 1.27
ODI at postoperative 24 months^*∗*^	28.00 ± 5.47	37.77 ± 11.49

^*∗*^ *P* < 0.05.
